# DNA Barcoding Medicinal Plant Species from Indonesia

**DOI:** 10.3390/plants11101375

**Published:** 2022-05-21

**Authors:** Ria Cahyaningsih, Lindsey Jane Compton, Sri Rahayu, Joana Magos Brehm, Nigel Maxted

**Affiliations:** 1 School of Biosciences, University of Birmingham, Edgbaston, Birmingham B15 2TT, UK; l.j.compton@bham.ac.uk (L.J.C.); joanabrehm@gmail.com (J.M.B.); n.maxted@bham.ac.uk (N.M.); 2 Research Center for Plant Conservation, Botanical Gardens and Forestry, National Research and Innovation Agency, Bogor 16122, Indonesia; srir005@brin.go.id

**Keywords:** DNA barcoding, medicinal plants, conservation, forensic, Indonesia

## Abstract

Over the past decade, plant DNA barcoding has emerged as a scientific breakthrough and is often used to help with species identification or as a taxonomical tool. DNA barcoding is very important in medicinal plant use, not only for identification purposes but also for the authentication of medicinal products. Here, a total of 61 Indonesian medicinal plant species from 30 families and a pair of ITS2, *matK*, *rbcL*, and *trnL* primers were used for a DNA barcoding study consisting of molecular and sequence analyses. This study aimed to analyze how the four identified DNA barcoding regions (ITS2, *matK*, *rbcL*, and *trnL*) aid identification and conservation and to investigate their effectiveness for DNA barcoding for the studied species. This study resulted in 212 DNA barcoding sequences and identified new ones for the studied medicinal plant species. Though there is no ideal or perfect region for DNA barcoding of the target species, we recommend *matK* as the main region for Indonesian medicinal plant identification, with ITS2 and *rbcL* as alternative or complementary regions. These findings will be useful for forensic studies that support the conservation of medicinal plants and their national and global use.

## 1. Introduction

Plant identification has formerly been formed using morphological characteristics that could be observed visually. Currently, DNA is also used to help species identification and to build bioinventories [1]. DNA barcoding was introduced by Hebert and colleagues in 2003 and involves the identification of species through universal, short, and standardized DNA regions [2]. DNA material for the barcoding can be obtained from living plants, herbarium specimens [3], and market products [4,5].

In plants, plastid DNA (*rbcL*, *matK*, *trnL*, and trnH-psbA regions) and nuclear DNA (ITS and ITS2 regions) are often used in DNA barcoding [6,7,8]. The *rbcL* and *matK* regions are recommended by the Consortium for the Barcode of Life (CBOL) as a standard two-locus barcode for global plant databases because of their species discrimination ability [8].

The process entails registering the DNA of identified species into a barcoding library and matching the DNA of unidentified species against the genetic data available in the library [6,9]. The library or the database can be accessed online for species identification and taxonomic clarification [10], namely through the NCBI GenBank (https://www.ncbi.nlm.nih.gov; accessed on 1 February 2022) [10] and the Barcode of Life Data (BOLD) (http://www.boldsystems.org; accessed on 1 February 2022) [11].

DNA barcoding has become an important taxonomic tool because of its accuracy, repeatability, and rapidity. It can also be used to identify species under legislative protection, or under threat of extinction, and to check the authenticity of biological products [6,9]. It is particularly powerful as identification is not influenced by the morphological diversity of species, growth phases, and environmental factors [12,13,14,15]. In the forensic field, even an inexperienced user is able to assign a taxonomic name to an unidentified plant specimen with relative ease [16,17]. It is an effective conservation tool as it is able to prevent substitution of important commercial species, protect species from theft [6,18], and help to define species richness in underexplored areas [6].

DNA barcoding is valuable in terms of medicinal plant (MP) species identification compared to traditional morphological identification for conservation and use, as it is able to identify species and ensure a genuine product rather than a substitute [6,18]. Identifying the plant correctly protects consumer rights [19], even with respect to small and damaged plant parts used in botanical forensics [10,20,21,22]. Several studies conducted on DNA barcoding of medicinal plants have indicated the effectiveness of ITS2 and *matK*. For example, these regions are able to distinguish *Rauvolfia serpentina* (L.) Benth. Ex Kurz, of which root extracts act as an antihypertensive drug from other species in the genus [5,23] and are able to authenticate *Eurycoma longifolia* Jack, of which all plant extracts (particularly roots) are a useful drug for cough, anticancer, and aphrodisiac activities [24]. *MatK* is also known to give the best identification for Philippine ethnomedicinal Apocynaceae [25]. However, DNA barcoding from only one specific sequence region has been applied for most medicinal plants. For example, the ITS2 region has been used as a DNA barcode for authenticating many medicinal plants, their relatives, and broader species [14,26], although it was found that this region could not authenticate all Chinese medicinal Bupleurum L. (Apiaceae) species [27]. For Indian medicinal plants (Ayurveda), the *rbcL* region has been used for DNA barcoding [19], while for medicinal plants of the Philippines, *rbcL*, *matK*, and *trnL*-F regions have been used based on their efficiency [28].

Indonesia is famous for its plant diversity and richness, particularly in medicinal plants and their uses [29,30,31]. Different forms of medicinal plants are used, regardless of being fresh or dried, for curing illness and disease [32]. Thus, the primary purpose of undergoing the barcoding process, apart from enriching the DNA barcoding database, is determining the identity of medicinal plants. DNA barcoding is an advanced technology for plant diversity inventories, and its high cost makes it both an issue and challenge for biodiversity conservation in Indonesia [33]. Nevertheless, DNA barcodes are useful for conservation and even for commercial purposes, and they will be widely used in the future as DNA sequencing technologies become simpler and cheaper [6]. This study aimed to assess how four different DNA barcoding regions (ITS2, *matK*, *rbcL*, and *trnL*) can aid 61 species identifications and conservation efforts, and investigate their effectiveness for DNA barcoding of Indonesian medicinal plants. The finding will allow for broader and more comprehensive use in the future with respect to medicinal plant conservation both nationally and globally.

## 2. Results and Discussions

### 2.1. Understanding the Use of DNA Barcoding for Indonesian Medicinal Plants

Of the 61 sampled Indonesian medicinal plants, 55 species are native to Indonesia (of which 29 are endemics), and six are introduced [34]. Some of the medicinal plants may need to be prioritized in terms of conservation, namely those assessed as threatened (VU, EN, CR) or near threatened (NT) according to the IUCN Red List [35], the 19 species listed in the CITES Appendices I, II, or III (UNEP-WCMC database) [36], and the 12 rare medicinal plants [37]. Two species were assessed as VU, namely *Aquilaria hirta* Ridl. [38] and *Etlingera solaris* (Blume) R.M.Sm. [39] and are considered to be facing a high extinction risk in the wild in the near future [40]. The 19 species listed in CITES II may become extinct if their trade is not controlled because they are collected from the wild and there is no sufficient data with respect to artificial propagation for commercial purposes [36]. Of the 61 sequence target species, 13 sequences were not found in BOLD, although their DNA sequence data was available in NCBI; a further 10 species did not have DNA sequences stored in either NCBI or BOLD. Detailed information for each of the 61 species is presented in Table 1.

**Figure 1:**
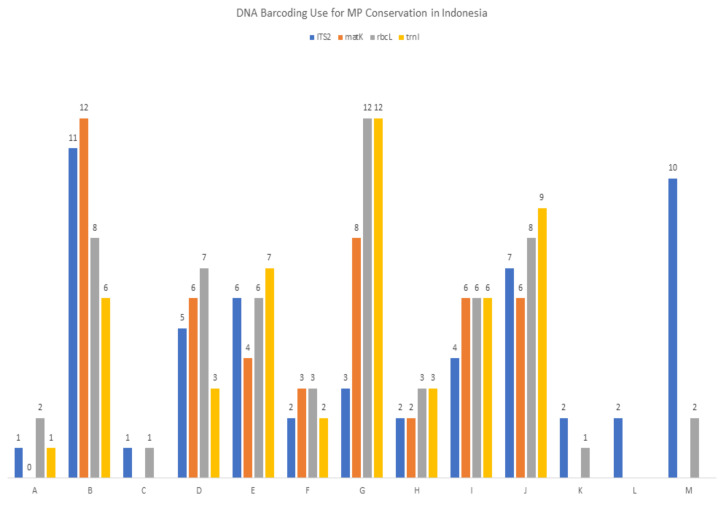
Summary of DNA barcoding use for medicinal plant (MP) conservation in Indonesia. Letters represent the DNA barcoding contribution of a species to the DNA bank data and its importance in conservation in the following order; A = new DNA barcoding and can strongly assist MP conservation; B = can strongly assist MP conservation; C = new DNA barcoding and can assist MP conservation; D = can assist MP conservation; E = new DNA bank data and new DNA barcoding and may strongly assist MP conservation; F = new DNA barcoding and may strongly assist MP conservation; G = may strongly assist MP conservation; H = new DNA bank data and new DNA barcoding and may assist MP conservation; I = new DNA barcoding and may assist MP conservation; J = may assist MP conservation; K = new DNA bank data and new DNA barcoding but sequences need to be clarified further; L = new DNA barcoding, but sequences need to be clarified further; M = sequences need to be clarified further.

The contribution of the DNA barcoding information from each species to DNA banks and to the correct identification of medicinal plants with high conservation status was classified using categories A–M, where category A denotes the contribution of new data to DNA banks and DNA barcoding information that can strongly assist MP conservation; at the opposite end of the spectrum, letter M denotes the least substantial contribution, where DNA barcoding needs to be clarified further before using it directly for identification. Figure 1 indicates how the four DNA barcodes are useful for the conservation and use of Indonesian medicinal plants with respect to the availability of their data in the DNA bank. The number of medicinal plant species per criteria are provided in Table A1. Sequences grouped in categories A-D can be of direct use to conservation efforts due to the correct identification of related medicinal plants. The A-B categories can be used in botanic forensics (in cases of medicinal plant adulteration and illegal trading) for medicinal plant identification [10,21,22,23,24], as the plants are listed as species that need to be prioritized in terms of conservation.There are 19 families of Indonesian medicinal plants consisting of 31 species, that could be identified accurately to the family level by DNA barcoding. Two major families of Indonesian medicinal plants that were successfully sequenced and correctly identified were Orchidaceae (13 sequences) and Apocynaceae (10 sequences). It is highlighted that correct identification was defined after the validation step, which is cross-checked to morphological identification result by taxonomists (indicated in the species identity card).

### 2.2. Understanding the Effectiveness of Each DNA Barcoding Region Used for Indonesian Medicinal Plants Identification

A total of 61 studied species were analyzed for DNA barcoding of four regions (ITS2, *matK*, *rbcL*, and *trnL*). There were some failures in DNA amplification and sequencing assembly, with the results of each step presented in Table 2.

The sequence quality is based on the easily done assembly of both the forward and reverse regions into a single consensus sequence (Table 2). When both forward and reverse sequences were available and were of good quality, obtaining the assembled consensus sequence was straightforward. If one direction of the sequence was mixed, then no assembly could occur and only the unidirectional sequence could be used. The *matK* region, which is known to have the lowest amplification success among the regions used for DNA barcoding [3,41], could only be amplified in 72% samples, compared with successful amplification in 83–98% samples for the other regions (Table 2). This is consistent with previous work indicating *matK* has a lower PCR success rate than *rbcL* for DNA amplification of Indonesian plants [42]. The PCR amplification failure likely occurred due to a high level of sequence variation within the *matK* regions complementary to the primers [43].

There were only 212 sequences of ITS2, *matK*, *rbcL*, and *trnL* obtained from 61 Indonesian medicinal plants instead of the expected 244 sequences resulting from the sequencing (Table A2). Each species was annotated with its key information, such as whether it is native, how the species became important to be conserved, and all generated sequences from ITS2, *matK*, *rbcL*, and *trnL* regions with identification results from BLAST, whether correct, ambiguous, correct at genus or family level, or incorrect.

### 2.3. Description of ITS2, matK, rbcL, and trnL Regions of Indonesian Medicinal Plants

**Figure 2:**
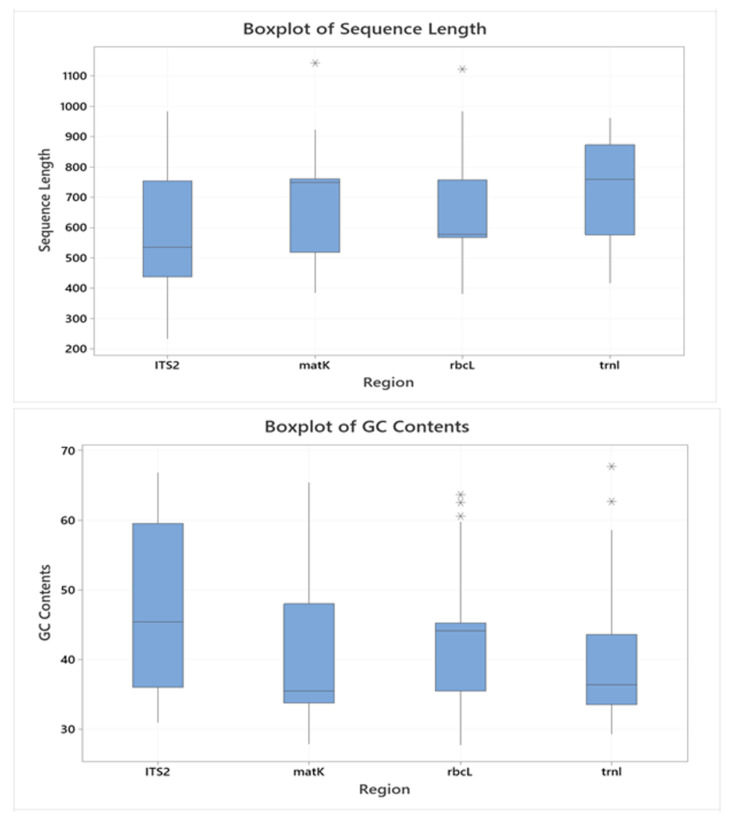
Box plots of the sequence length (**upper**) and GC content (**lower**) of ITS2, *matK*, *rbcL*, and *trnL* of Indonesian medicinal plants.

The descriptive statistics of the sequence regions ITS2, *matK*, *rbcL*, and *trnL* are portrayed in Figure 2. The minimum and maximum lengths (bp) of ITS2, *matK*, *rbcL*, and *trnL* regions varied between 233–984, 384–1142, 382–1122, and 416–962, respectively, for all studied species; the average lengths of each region were 591.2, 676.9, 636.1, and 735.8, respectively. The range of GC Content (%) for ITS2, *matK*, *rbcL*, and *trnL* regions varied between 30.94–66.83, 27.86–65.43, 27.72–63.64, and 29.26–67.74, respectively, for all Indonesian medicinal plant species, whilst the average GC contents were 48.34, 41.64, 43.52, and 39.10, respectively.

**Figure 3:**
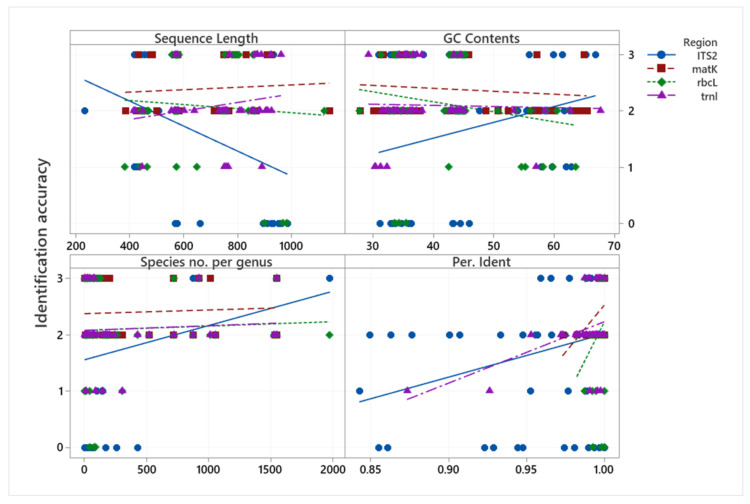
Scatterplot of identification accuracy vs. sequence length (bp), GC Content (%), species number per genus, and percentage of identity. Scale 0–3 represents the identification accuracy (0 = incorrect, 1 = correct at the family level, 2 = correct at the genus level, 3 = correct at the species level).

The relationships between MP species identification accuracy and sequence length (bp), GC content (%), species number per genus, and percentage of identity are presented in Figure 3. With respect to sequence length, the longer the ITS2 and *rbcL* sequence regions, the lower the identification accuracy, while the other regions indicated no such relationship. With respect to GC content (%), all regions except ITS2 tended to be less accurate for identification when the GC content increased. In terms of species number per genus, *matK*, *rbcL*, and *trnL* regions all tended to have no correlation with the species number per genus, but the ITS2 sequence region was more accurate in identification when the species number per genus was higher. However, this result will depend on the available DNA information in the data bank. All regions indicated a positive relationship of percentage identity (through a BLASTN search) with identification accuracy.

Among the sequence regions produced for Indonesian medicinal plants, ITS2 generally had the lowest minimum length, smallest average sequence, and highest GC content (Figure 1 and Figure 2) and hence gives the highest efficiency of identification, with only a short DNA sequence needed for correct identification. After ITS2, *matK* follows second with respect to having the smallest average sequence length. A short DNA sequence may make the process of DNA barcoding technically easier and more economical from extraction to sequencing, as Kress and colleagues suggested [44]. Meanwhile, in terms of GC content (%), only ITS2 had higher identification accuracy when the GC content increased. In some plant DNA sequences, GC content has a positive correlation with exon sites, i.e., the coding regions [45]. This might mean that the longer the exons, the higher the GC content; thus, DNA regions with high GC content are expected to have more accurate identification.

### 2.4. Identification of Indonesian Medicinal Plants Using Sequences of Their ITS2, matK, rbcL, and trnL Regions

Identification of the sequence regions resulting from the BLAST method that have been matched with samples morphologically identified are presented in Table 3. The highest correct identification in the set of medicinal plant species was reached by the *matK* region, followed by ITS2 and *rbcL*, although the percentage values among them were not significantly different (i.e., 31.15% compared to 29.51%). In contrast, *trnL* had the lowest correct identification, approximately 15% lower than that of *matK*. The highest incorrect identification was reached by the ITS2 region, followed by the *rbcL* region. Overall, the most accurate of the four regions was *matK* because it has the highest identification rate at the species level, lowest at the family level, and resulted in no incorrect identifications [3,41,42].

Some ambiguous (correct at the genus and family level) and incorrect identification of Indonesian medicinal plants occurred. This might have happened because the world plant data has more than 1.2 million species names [34], while the DNA barcoding data for plants contains only 234,692 barcodes and only 5942 plants are recorded from Indonesia (http://www.boldsystems.org; accessed on 6 February 2020). As such, the available DNA bank to be cross-checked with the samples is far from complete.

**Figure 4:**
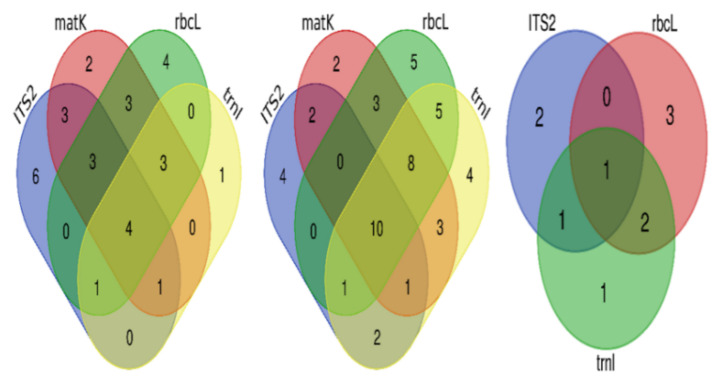
Venn diagrams for correct identification of species at different taxonomic levels. From left to right: at the species level, at the genus level, and at the family level.

The correct identification of unique species by singular regions and by combinations of regions can be visualized in the Venn diagrams (Figure 4). ITS2 was the most accurate region with unique correct identification, followed by *rbcL*, *matK,* and *trnL*. A combination of three regions gave the same number of unique correct identifications, and a combination of all gave the highest correct identification. With respect to unique correct identification at the genus level, *rbcL* gave the most accurate identification, followed by ITS2, *trnL*, and finally *matK*. A combination of *matK*, *rbcL*, and *trnL* gave the best unique accurate identification compared to the other three combinations, and the combination of all gave the largest number of unique species among all possibilities. The highest unique correct species at the family level were obtained by using *rbcL*, then ITS2, and finally *trnL*.

As presented in Table 4, the overall averages of the barcoding regions describing the genetic distance between the two compared species were very similar to one another, i.e., above 1.1% and below 1.2%, except for ITS2, which indicated an average of 1.29%. The lower the taxon unit relation, the lower the percentage, while the higher the taxon unit relation, the higher the percentage. Only the minimum distance of the *matK* region could describe species in the same genera. Nevertheless, the maximum distance of each region describes the highest level of the different species in a family. In principle, the genetic distance of interspecific related species (within the genus level and above) should be greater than that of the intraspecific species (within species level). It can be stated that more genetic distance lies between two different species with a different family than two different species with the same family. Species within the same genus have the least genetic distance.

The percentage of the sequences identified for each of the regions (ITS2, *matK*, *rbcL*, and *trnL*) was directly proportional to the accuracy of the identification. The higher the percentage, the more accurate the identification. *MatK* could correctly lead to identification of species with the highest percentages, followed by *rbcL* and ITS2 (Table 2). Only the *matK* region could differentiate species at the same genus level and species in different families compared to other regions. In contrast, ITS2 could not differentiate all species distances appropriately (Table 4).

In addition, it should be considered that using BLAST in a DNA barcoding study with any regions/primers is a basic step in identifying species [25,26,27,28,42]. BLAST analysis is the approach to the most similar species, and it depends on the species information stored in DNA bank. Therefore, the validation step to confirm the most accurate or most possible species is still required. When the used samples were clear species [42] like in this study, morphological identification by the experts was used, but when the samples were unable to be identified morphologically due to damage or derivate form or/and lack of taxonomic expert, generating a phylogenetic tree amongst medicinal plant groups such as a neighbor-joining (NJ) tree [23,25,26,42], maximum parsimony (MP), and maximum likelihood (ML) [42], and even analyzing chemical compound products [24] might be needed.

Considering Hollingsworth and colleagues’ findings with respect to DNA barcoding, it could serve two purposes. The first would be to provide information into the species-level taxon unit, and the second would be to help identify an unknown specimen to a known species. Thus, all the regions tested are valuable, depending on the purpose [43]. We emphasize that having a phylogenetic tree in the barcoding study would be beneficial, particularly when experts assume the medicinal plants are unidentified or a cryptic species. Thus, identification, authentication, and even conservation plan and action can be properly defined and applied.

## 3. Materials and Methods

### 3.1. Plant Samples and Literature Survey

This study used 61 different species of medicinal plants belonging to 30 families and 50 genera (Table 1). Plant samples were collected from a living collection with written permission from botanic gardens, including Bogor Botanic Gardens and Cibodas Botanic Gardens in Indonesia, and Hortus Botanicus Leiden in the Netherlands. All species had been taxonomically identified using morphological features as viewed on their identity card. Their scientific names were cross-checked online using POWO (2022) [34]. A leaf sample was collected from each species, except for *Alstonia scholaris* (L.) R. Br. and *Spondias malayana* Kosterm, from which bark samples were taken. This was due to *A. scholaris* and *S. malayana* Kosterm being high trees with unreachable leaves. Each sample (approximately 25 g) was collected and stored in a teabag with silica gel [46,47,48].

A literature study was conducted to collect all scientific information with respect to each of the sampled plant species, which can help the conservation status of every species. Information about available DNA data—i.e., whether the species already had DNA barcoding or genetic information that could be accessed from DNA banks—was identified using BOLD [11] and NCBI [10]. Data on species origin, including whether the species are native or introduced to Indonesia, and, if native, whether they are endemic, were collected from POWO (http://www.plantsoftheworldonline.org; accessed on 1 February 2022) [34]. Threatened species status was collected from the IUCN Red List of Threatened Species (https://www.iucnredlist.org; accessed on 6 February 2022), with species classified as Vulnerable (VU), Endangered (EN), Critically Endangered (CR), Extinct in The Wild (EW), or Extinct (EX) [35]. Global legislation regulating trade, i.e., based on whether the species is included in CITES Appendices I, II, or III, was collected from the UNEP-WCMC Checklist of CITES species (https://checklist.cites.org; accessed on 1 February 2022) [36]. The information on rare medicinal plants, was compiled from the Indonesian Biodiversity Strategy and Action Plan (IBSAP) National Document [37]. Endemic plants or plants mentioned in the IUCN Red List, CITES Appendices I, II, or III, endemic, and priority lists were considered to be important species that need to be prioritized for conservation [49].

### 3.2. Molecular Analysis

Molecular analysis was performed at the University of Guelph laboratory, Canada. The barcoding method involves genomic DNA extraction, DNA amplification, and DNA sequencing, and taxonomic identification against available DNA banks. For DNA extraction, genomic DNA was extracted from plant samples using the Maxwell^®^ RSC Purefood GMO and Authentication Kit and the Maxwell^®^ RSC Instrument (Promega). For DNA amplification, primers targeting the ITS2, *matK*, *rbcL*, and *trnL* genes of plants were used to amplify the DNA (Table 5). Each PCR reaction mix (25 μL) contained 1x HotStarTaq master mix (Qiagen), 0.4 μM of each (forward and reverse) primers, 0.15 μg of BSA and 2 μL of template DNA. PCR thermal cycling was conducted by using a GeneAmpTM PCR System 9700 (Applied Biosystems, Waltham, MA, USA). The PCR cycling conditions were as follows: 95 °C for 10 min for DNA denaturation, 45 cycles of 95 °C for 15 sec for DNA annealing with the primer, followed by 55 °C for 30 sec and 72 °C for 1 min for DNA extension, and finally 72 °C for 7 min.

PCR products were visualized on 2% agarose gels to check whether DNA amplification was successful. PCR products were then purified using a NucleoFast^®^ 96 PCR clean-up kit (Macherey-Nagel). The purified PCR fragments were sequenced bidirectionally, using the same primers as for the PCR, with the help of an ABI 3730 Genetic Analyzer (Applied Biosystems). The retrieved sequences were analyzed using ABI Prism^TM^ Sequencing Analysis software (Applied Biosystems) to obtain a consensus sequence (Q > 20) for each sample.

### 3.3. Sequence Analyses and Data Interpretation

For each sample, the consensus sequence was compared with the nucleotide sequences in the BOLD species ID engine and the NCBI GenBank using BLASTN (https://blast.ncbi.nlm.nih.gov; accessed on 7 January 2022) [52] with the program selection as “Highly Similar Sequences (Megablast)” [53] for taxonomic identification. When no result was obtained from Megablast due to the sequence being too short, the sequence was queried with the program selection as, “Somewhat similar sequences (nBlast) for an alternative”.

PCR amplification, sequencing, and identification success rates were calculated as percentages. Only one best-matched species was selected from the BLASTN identification that is approached from the most similar sequence species recorded in DNA bank. Where there was more than a single match, the best-matched species was selected as the one with the lowest E value and the highest coverage; otherwise, any species was the closest-related species to the query (species). The results were then validated with studied medicinal species’ ID from botanical gardens where they have been morphologically identified by taxonomic expert.

The BLAST identification results were the initial step to identify species with DNA barcoding [25,26,27,28,42]. It was considered to be the correct species if the highest percentage of identification referred to the right species, i.e., when the species name from sequence identification matched the morphologically identified species. Otherwise, when the sequence was identified as a different species within a genus or a different species within a family, the result was considered to be an ambiguous species or genus. Ambiguous identifications were counted as correct identification, as per the study by Amandita et al. [42]. Sequences with an identification percentage of 99% or more were included in the novel sequence data for specific DNA barcoding for a species. Novel sequence data will be deposited in the GenBank database to assist in future identification.

Descriptive, statistical, and scatter plot analyses were used to gain understanding of the ITS2, *matK*, *rbcL*, and *trnL* regions and the relationship between factors in the BLAST analysis, with the identification being completed using the MINITAB Statistical Software.

In addition, Venn diagrams generated by Bioinformatics and Evolutionary Genomics (http://bioinformatics.psb.ugent.be/cgi-bin/liste/Venn/calculate_venn.htpl; accessed on 2 January 2022) were used to depict how many species were correctly identified by singular regions and by multiple combinations of regions, whether or now there was a correct identification within species, genus, or family level. Information about the species number per genus was obtained from POWO [34].

Sequence alignments were performed using the Muscle program. The nucleotide composition of all sequences obtained from the ITS2, *matK*, *rbcL*, and *trnL* regions were computed, and their genetic distances were calculated with Kimura 2 parameters (K2P) [54]. The K2P pairwise genetic distance is the percentage of nucleotide sequence divergence that was used by Hebert and colleagues [2]. All analyses were performed with the Molecular Evolutionary Genetics Analysis (MEGA X) software [55].

All the medicinal plant species information collected was analyzed and interpreted according to the use of the data in DNA barcoding with respect to conservation. Any correct identification can be used for DNA barcoding for related species and can be subsequently helpful for medicinal plant conservation, although the DNA barcoding can only be used for identification at species level and cannot estimate variation within species [56]. Any ambiguous identification can be used as an approach to species identification and thus may also be valuable for medicinal plant conservation.

Any new sequence or new DNA barcoding that is not available in NCBI or BOLD constitutes novel data. Species included in at least one of the following categories: IUCN Red List [40], CITES Appendixes I, II, or III [36], rare medicinal plants species [37], or Native and Endemic species [34] would require DNA barcoding more urgently than the non-listed species. Therefore the species were categorized in priority order A-M as follows: new DNA barcoding and can strongly assist medicinal plant (MP) conservation (A), can strongly assist MP conservation (B), new DNA barcoding and can assist MP conservation (C), can assist MP conservation (D), new to DNA bank data and new DNA barcoding and may strongly assist MP conservation (E), new DNA barcoding and may strongly assist MP conservation (F), may strongly assist MP conservation (G), new to DNA bank data and new DNA barcoding and may assist MP conservation (H), new DNA barcoding and may assist MP conservation (I), may assist MP conservation (J), new to DNA bank data and new DNA barcoding but sequences need to be clarified further (K), new DNA barcoding but sequences need to be clarified further (L) and sequences need to be clarified further (M).

## 4. Conclusions

Based on the results of this study, we conclude that no single region is perfectly ideal for DNA barcoding. Nonetheless, according to the observed criteria, we recommend *matK* as the core DNA barcoding region for Indonesian medicinal plant identification. In addition, due to its unique correct species identification, we recommended the ITS2 and *rbcL* regions as alternative or complementary regions to the core barcoding DNA using *matK*. DNA barcoding for 33 Indonesian medicinal plant species was provided; of these 33 species, 21 species were newly DNA barcoded; of these 21 species, three contributed novel DNA barcoding data to DNA bank. In the future, this guide and associated data will facilitate a means to identify Indonesian medicinal plants, particularly those that need to be conserved strongly, to assure a valid species rather than a substitute in herbal medicines and to prevent illegal trade.

## Figures and Tables

**Table 1:** The Indonesian medicinal plants (n = 61) used in this study with related information from literature study.

No.	Species	Author	Family.	N/I	Important Sp.	Sp. No. per Genus	BOLD (NCBI)Database
1	*Justicia gendarussa*	Burm.f.	Acanthaceae	N	No	921	yes
2	*Staurogyne elongata*	(Nees) Kuntze	Acanthaceae	N	No	148	yes
3	*Pangium edule*	Reinw.	Achariaceae	N	Yes (P)	1	yes
4	*Spondias malayana*	Kosterm.	Anacardiaceae	N	No	19	no (yes)
5	*Toxicodendron succedaneum*	(L.) Kuntze	Anacardiaceae	I	No	27	yes
6	*Ancistrocladus tectorius*	(Lour.) Merr.	Ancistrocladaceae	N	No	21	yes
7	*Anaxagorea javanica*	Blume	Annonaceae	N	Yes (P)	25	no (yes)
8	*Dasymaschalon dasymaschalum*	(Blume) I.M.Turner	Annonaceae	N	No	27	yes
9	*Alstonia macrophylla*	Wall. Ex. G.Don	Apocynaceae	N	No	44	yes
10	*Alstonia scholaris*	(L.) R. Br.	Apocynaceae	N	Yes (P)		yes
11	*Alyxia reinwardtii*	Blume	Apocynaceae	N	Yes (P)	106	yes
12	*Hoya diversifolia*	Blume	Apocynaceae	N	No	521	no (yes)
13	*Rauvolfia serpentina*	(L.) Benth. ex Kurz	Apocynaceae	N	Yes (II)	74	yes
14	*Aglaonema commutatum*	Schott	Araceae	N	No	22	no (yes)
15	*Trevesia burckii*	R.Br.	Araliaceae	N	No	8	yes (yes)
16	*Cibotium barometz*	(L.) J.Sm.	Cibotiaceae	N	Yes (II)	10	yes
17	*Decalobanthus mammosus*	(Lour.) A.R.Simoes & Staples	Convolvulaceae	I	No	13	no (yes)
18	*Erycibe malaccensis*	C.B. Clarke	Convolvulaceae	N	No	70	no (no)
19	*Rhododendron macgregoriae*	F. Muell.	Ericaceae	N	Yes (E)	1057	no (no)
20	*Acalypha grandis*	Benth.	Euphorbiaceae	N	No	428	no (no)
21	*Euphorbia tirucalli*	L.	Euphorbiaceae	I	Yes (II)	1976	yes
22	*Millettia sericea*	(Vent.) Benth.	Fabaceae	N	No	187	yes
23	*Parkia timoriana*	(DC.) Merr.	Fabaceae	N	No	40	yes
24	*Phanera fulva*	(Korth.) Benth.	Fabaceae	N	Yes (E)	90	no (no)
25	*Orthosiphon aristatus*	(Blume) Miq.	Lamiaceae	N	No	44	yes
26	*Premna serratifolia*	L.	Lamiaceae	N	No	131	yes
27	*Vitex glabrata*	Gaertn.	Lamiaceae	N	No	203	yes
28	*Cinnamomum rhynchophyllum*	Miq.	Lauraceae	N	No	241	no (yes)
29	*Ficus deltoidea*	Jack	Moraceae	N	Yes (P)	874	yes
30	*Myristica succedanea*	Blume	Myristicaceae	N	Yes (E)	175	no (no)
31	*Nepenthes ampullaria*	Jack	Nepenthaceae	N	Yes (P, II)	165	yes
32	*Nepenthes gracilis*	Korth.	Nepenthaceae	N	Yes (P, II)		yes
33	*Nepenthes mirabilis*	(Lour.) Druce	Nepenthaceae	N	Yes (P, II)		yes
34	*Nepenthes reinwardtiana*	Miq.	Nepenthaceae	N	Yes (P, E, II)		yes
35	*Acriopsis liliifolia* var. *liliifolia*	(J.Koenig) Ormerod	Orchidaceae	N	Yes (P, II)	10	no (yes)
36	*Cymbidium aloifolium*	(L.) Sw.	Orchidaceae	N	Yes (P, II)	74	yes
37	*Cymbidium ensifolium*	(L.) Sw.	Orchidaceae	I	Yes (II)		yes
38	*Dendrobium crumenatum*	Sw.	Orchidaceae	N	Yes (P, II)	1547	yes
39	*Dendrobium purpureum*	Roxb.	Orchidaceae	N	Yes (P, E, II)		no (no)
40	*Dendrobium salaccense*	(Blume) Lindl.	Orchidaceae	N	Yes (P, II)		yes
41	*Grammatophyllum speciosum*	Blume	Orchidaceae	N	Yes (P, II)	13	yes
42	*Nervilia concolor*	(Blume) Schltr.	Orchidaceae	N	Yes (P, II)	77	yes
43	*Nervilia plicata*	(Andrews) Schltr.	Orchidaceae	N	Yes (P, II)		yes
44	*Oberonia lycopodioides*	(J.Koenig) Ormerod	Orchidaceae	N	Yes (P, II)	305	no (no)
45	*Strongyleria pannea*	(Lindl.) Schuit., Y.P.Ng & H.A.Pedersen	Orchidaceae	N	Yes (P, II)	4	no (yes)
46	*Galearia filiformis*	(Blume) Boerl.	Pandaceae	N	Yes (E)	5	yes
47	*Benstonea affinis*	(Kurz) Callm. & Buerki	Pandanaceae	N	No	61	yes
48	*Phyllanthus oxyphyllus*	Miq.	Phyllanthaceae	N	No	1016	yes
49	*Ardisia complanata*	Wall.	Primulaceae	N	No	719	no (no)
50	*Ardisia crenata*	Sims	Primulaceae	I	No		yes
51	*Ventilago madraspatana*	Boerl.	Rhamnaceae	N	No	41	no (yes)
52	*Psychotria montana*	Blume	Rubiaceae	N	No	1531	no (yes)
53	*Lunasia amara*	Blanco	Rutaceae	N	Yes (P)	1	yes
54	*Melicope lunu-ankenda*	(Gaertn.) T.G. Hartley	Rutaceae	N	No	241	no (yes)
55	*Kadsura scandens*	(Blume) Blume	Schisandraceae	N	Yes (P)	17	yes
56	*Smilax calophylla*	Wall. ex A.DC.	Smilacaceae	N	No	262	yes
57	*Smilax zeylanica*	L.	Smilacaceae	N	Yes (P)		yes
58	*Aquilaria hirta*	Ridl.	Thymelaeaceae	N	Yes (P, VU)	21	no (yes)
59	*Amomum hochreutineri*	Valeton	Zingiberaceae	N	Yes (E)	102	no (no)
60	*Etlingera solaris*	(Blume) R.M.Sm.	Zingiberaceae	N	Yes (E, VU)	143	no (no)
61	*Meistera aculeata*	(Roxb.) Skornick. & M.F. Newman	Zingiberaceae	N	No	41	no (yes)

Note: Scientific names (1st and 2nd columns were collected from POWO (2022); Species: R for rare medicinal plant (MP), E for endemic to Indonesia, VU for Vulnerable (IUCN Red List), P for Priority, and II for CITES Appendix II; N = Native, I = Introduced.

**Table 2:** Success or failure in each DNA barcoding step.

Observed Parameter	ITS2 (%)	*matK* * (%)	*rbcL* (%)	*trnL* (%)
No PCR amplicon obtained	1.64	27.87	1.64	16.39
Mixed sequences—no use	8.20	0	1.64	3.28
Sequence provided	90.16	72.13	96.72	80.33
Assembled consensus sequence	88.52	65.57	96.72	73.77
Unidirectional sequence	1.64	6.56	0	6.56

* 4 *matK* regions with the second primer excluded.

**Table 3:** Identification success rates of each region through the BLAST method after validating with the species name from morphologicy identification.

Identification Measure	Region			
	ITS2 (%)	*matK* * (%)	*rbcL* (%)	*trnL* (%)
Correct identification at species level	29.51	31.15	29.51	16.39
Correct identification at genus level	32.79	47.54	52.46	55.74
Correct identification at family level	6.56	0	9.84	8.20
Incorrect identification	22.95	0	4.92	0

* 4 *matK* regions with the second primer excluded.

**Table 4:** K2P pairwise genetic distances (%) of each region at different species levels.

Region	Observation	Value (%)	Related Species
ITS2	Overall average	1.29503	
	Minimum distance	0.00440	*Nepenthes reinwardtiana* and Nervilia concolor ***
	Maximum distance	2.70903	*Erycibe malaccensis* and Acalypha grandis ***
*matK*	Overall average	1.12567	
	Minimum distance	0.00615	*Nepenthes mirabilis* and N. ampullaria *
	Maximum distance	2.62368	*Nepenthes reinwardtiana* and Parkia timoriana ***
*rbcL*	Overall average	1.19148	
	Minimum distance	0.00350	*Amomum hochreutineri* and Etlingera solaris **
	Maximum distance	2.62587	*Phyllanthus oxyphyllus* and Galearia filiformis ***
*trnL*	Overall average	1.11310	
	Minimum distance	0.02887	Alstonia scholaris and Rauvolfia serpentina **
	Maximum distance	2.59858	Millettia sericea and Cymbidium aloifolium ***

Notes: *: MP species in the same genera; **: MP species in the same family; ***: MP species in the different family.

**Table 5:** Primers used for amplification of DNA regions of ITS2, *matK*, *rbcL*, and *trnL*.

Gene Region	Name	Sequence	Reference
*rbcL*	*rbcL*a-F	ATGTCACCACAAACAGAGACTAAAGC	[50]
	*rbcL*a-R	GTAAAATCAAGTCCACCRCG	
*matK*	*matK*472F	CCCRTYCATCTGGAAATCTTGGTTC	[41]
	*matK*1248R	GCTRTRATAATGAGAAAGATTTCTGC	
*matK* ^a^	*matK*xF	TAATTTACGATCAATTCATTC	[23]
	*matK*5R	GTTCTAGCACAAGAAAGTCG	
ITS2	ITS2F	ATGCGATACTTGGTGTGAAT	[51]
	ITS3R	GACGCTTCTCCAGACTACAAT	
*trnL*	*trnL-F*	ATTTGAACTGGTGACACGAG	[7]
	*trnL*-c	CGAAATCGGTAGACGCTACG	

Note: *matK* ^a^ is an alternative to *matK* that is used when the PCR reaction fails to have an amplificon. F denotes the forward primer sequence and R is the reverse primer sequence.

## Data Availability

All data resulting from this study has been stored and could be accessed at http://www.boldsystems.org under Project-MPIN DNA BARCODING STUDY OF MEDICINAL PLANTS OF INDONESIA FOR ASSISTING THEIR CONSERVATION AND USE.

## Appendix A

**Table A1:** DNA barcoding regions used for medicinal plant (MP) conservation in Indonesia.

DNA Barcoding Use for MP Conservation in Indonesia	ITS2	*matK*	*rbcL*	*trnL*
**A. new DNA barcoding and can strongly assist MP conservation**	**1**	**1**	**2**	**1**
*Anaxagorea javanica*				1
*Aquilaria hirta*			1	
*Strongyleria pannea*	1	1	1	
**B. can strongly assist MP conservation**	**11**	**12**	**8**	**6**
*Alstonia scholaris*	1	1	1	1
*Alyxia reinwardtii*	1	1	1	
*Cymbidium aloifolium*	1	1	1	
*Dendrobium crumenatum*	1	1		
*Dendrobium salaccense*		1	1	1
*Euphorbia tirucalli*	1			
*Ficus deltoidea*	1			
*Galearia filiformis*		1	1	1
*Kadsura scandens*	1			
*Lunasia amara*	1	1		1
*Nepenthes gracilis*		1		
*Nepenthes reinwardtiana*	1	1		
*Nervilia plicata*		1	1	1
*Pangium edule*		1	1	
*Parkia timoriana*	1			
*Rauvolfia serpentina*	1	1	1	1
**C. new DNA barcoding and can assist MP conservation**	**1**		**1**	
*Aglaonema commutatum*			1	
*Meistera aculeata*	1			
**D. can assist MP conservation**	**5**	**6**	**7**	**3**
*Alstonia macrophylla*		1	1	
*Ancistrocladus tectorius*	1		1	1
*Ardisia crenata*		1	1	
*Dasymaschalon dasymaschalum*			1	
*Justicia gendarussa*	1	1	1	1
*Orthosiphon aristatus*	1			
*Phyllanthus oxyphyllus*	1	1		
*Premna serratifolia*			1	
*Toxicodendron succedaneum*	1	1	1	1
*Vitex glabrata*		1		
**E. new to DNA bank data and new DNA barcoding and may strongly assist MP conservation**	**6**	**4**	**6**	**7**
*Amomum hochreutineri*	1		1	1
*Dendrobium purpureum*	1	1	1	1
*Etlingera solaris*	1		1	1
*Myristica succedanea*		1	1	1
*Oberonia lycopodioides*	1	1	1	1
*Phanera fulva*	1			1
*Rhododendron macgregoriae*	1	1	1	1
**F. new DNA barcoding and may strongly assist MP conservation**	**2**	**3**	**2**	**2**
*Acriopsis liliifolia* var. *liliifolia*	1	1	1	1
*Anaxagorea javanica*		1	1	
*Aquilaria hirta*	1	1		1
**G. may strongly assist MP conservation**	**3**	**8**	**12**	**12**
*Alyxia reinwardtii*				1
*Cibotium barometz*			1	
*Cymbidium aloifolium*				1
*Cymbidium ensifolium*	1	1		
*Dendrobium crumenatum*			1	
*Dendrobium salaccense*	1			
*Euphorbia tirucalli*			1	
*Ficus deltoidea*		1	1	1
*Grammatophyllum speciosum*		1	1	1
*Kadsura scandens*		1	1	1
*Lunasia amara*			1	
*Nepenthes ampullaria*		1	1	1
*Nepenthes gracilis*			1	1
*Nepenthes mirabilis*	1	1	1	1
*Nepenthes reinwardtiana*			1	1
*Nervilia concolor*				1
*Pangium edule*				1
*Parkia timoriana*		1		1
*Smilax zeylanica*		1	1	
**H. new to DNA bank data and new DNA barcoding and may assist MP conservation**	**2**	**2**	**3**	**3**
*Acalypha grandis*			1	1
*Ardisia complanata*	1	1	1	1
*Erycibe malaccensis*	1	1	1	1
**I. new DNA barcoding and may assist MP conservation**	**4**	**6**	**7**	**6**
*Aglaonema commutatum*		1		1
*Cinnamomum rhynchophyllum*		1	1	1
*Decalobanthus mammosus*			1	
*Hoya diversifolia*	1	1	1	1
*Meistera aculeata*			1	
*Melicope lunu-ankenda*	1	1	1	1
*Psychotria montana*	1	1	1	1
*Spondias malayana*	1			
*Ventilago madraspatana*		1	1	1
**J. may assist MP conservation**	**7**	**6**	**8**	**9**
*Alstonia macrophylla*	1			1
*Ancistrocladus tectorius*		1		
*Ardisia crenata*	1			1
*Benstonea affinis*		1	1	1
*Dasymaschalon dasymaschalum*		1		1
*Millettia sericea*	1	1	1	1
*Orthosiphon aristatus*			1	
*Phyllanthus oxyphyllus*			1	1
*Premna serratifolia*	1			
*Smilax calophylla*			1	
*Staurogyne elongata*	1	1	1	1
*Trevesia burckii*	1	1	1	1
*Vitex glabrata*	1		1	1
**K. new to DNA bank data and new DNA barcoding, but sequences need to clarify further (K)**	**2**		**1**	
*Acalypha grandis*	1			
*Myristica succedanea*	1			
*Phanera fulva*			1	
**L. new DNA barcoding, but sequences need to clarify further**	**2**			
*Aglaonema commutatum*	1			
*Ventilago madraspatana*	1			
**M. new DNA barcoding and may strongly assist MP conservation**	**10**		**2**	
*Benstonea affinis*	1			
*Cibotium barometz*	1			
*Dasymaschalon dasymaschalum*	1			
*Galearia filiformis*	1			
*Grammatophyllum speciosum*	1			
*Nervilia concolor*	1		1	
*Nervilia plicata*	1			
*Pangium edule*	1			
*Parkia timoriana*			1	
*Smilax calophylla*	1			
*Smilax zeylanica*	1			

**Table A2:** Summary of DNA barcoding result per species.

No.	Species [38]	Author	Fam.	Region	Max Score	Total Score	Query Cover	E Value	Per. Ident	Best Matched Species	Sum.	Notes
1	*Justicia gendarussa*	Burm.f.	Acanth.	ITS2	562	562	0.73	5.00E-156	0.9968	*Justicia gendarussa*	c	
				*matK*	1330	1330	0.96	0	0.9986	*Justicia gendarussa*	c	
				*rbcL*	1055	1055	0.97	0	1	*Justicia gendarussa*	c	
				*trnL*	1487	1487	0.92	0	0.9975	*Justicia gendarussa*	c	
2	*Staurogyne elongata*	(Nees) Kuntze	Acanth.	ITS2	597	597	0.89	1.00E-166	0.9526	*Ophiorrhiziphyllon macrobotryum*	a **	
				*matK*	1273	1273	0.97	0	0.9821	*Staurogyne concinnula*	a *	
				*rbcL*	939	939	0.91	0	0.9923	*Staurogyne concinnula*	a *	
				*trnL*	1013	1427	0.99	0	0.9732	*Staurogyne trinitensis*	a *	
3	*Pangium edule*	Reinw.	Achari.	ITS2	163	163	0.15	1.00E-35	0.9286	*Celastraceae* sp.	i	
				*matK*	1387	1387	1	0	0.9974	*Pangium edule*	c	
				*rbcL*	972	972	0.91	0	1	*Pangium edule*	c	
				*trnL*	1158	1741	0.98	0	0.982	*Ryparosa kurrangii*	a *	
4	*Spondias malayana*	Kosterm.	Anacardi.	ITS2	636	636	1	3.00E-178	0.9332	*Spondias tuberosa*	a *	
5	*Toxicodendron succedaneum*	(L.) Kuntze	Anacardi.	ITS2	660	660	0.75	0	1	*Toxicodendron succedaneum*	c	
				*matK*	1452	1452	0.99	0	1	*Toxicodendron succedaneum*	c	
				*rbcL*	1038	1038	0.97	0	1	*Toxicodendron succedaneum*	c	
				*trnL*	1598	1598	1	0	1	*Toxicodendron succedaneum*	c	1/7 is a *
6	*Ancistrocladus tectorius*	(Lour.) Merr.	Ancistroclad.	ITS2	774	774	1	0	0.9953	*Ancistrocladus benomensis*	c	1/3 is a *
				*matK*	1387	1387	1	0	0.9987	*Ancistrocladus heyneanus*	a *	
				*rbcL*	1053	1053	1	0	1	*Ancistrocladus tectorius*	c	
				*trnL*	1663	1663	1	0	0.9903	*Ancistrocladus tectorius*	c	
7	*Anaxagorea javanica*	Blume	Annon.	*matK*	1502	1502	0.97	0	0.9928	*Anaxagorea luzonensis*	a *	
				*rbcL*	1013	1013	0.94	0	1	*Anaxagorea luzonensis*	a *	
				*trnL*	1423	1423	1	0	1	*Anaxagorea javanica*	c	
8	*Dasymaschalon dasymaschalum*	(Blume) I.M.Turner	Annon.	ITS2	237	237	0.38	3.00E-58	0.9474	*Acer palmatum*	i	
				*matK*	1382	1382	1	0	0.9947	*Dasymaschalon clusiflorum*	a *	
				*rbcL*	1020	1020	0.97	0	1	*Desmos dasymaschalus*	c	
				*trnL*	1565	1565	0.95	0	0.9965	*Dasymaschalon megalanthum*	a *	
9	*Alstonia macrophylla*	Wall. Ex. G.Don	Apocyn.	ITS2	763	763	0.98	0	0.9976	*Alstonia scholaris*	a *	
				*matK*	1386	1386	1	0	0.9987	*Alstonia macrophylla*	c	
				*rbcL*	857	857	1	0	0.9876	*Alstonia scholaris*	c	13/14 is a * with the same coverage
				*trnL*	1557	1557	1	0	0.9908	*Alstonia scholaris*	a *	
10	*Alstonia scholaris*	(L.) R. Br.	Apocyn.	ITS2	457	457	0.62	3.00E-124	0.9772	*Alstonia scholaris*	c	
				*matK*	1380	1380	1	0	0.9987	*Alstonia yunnanensis*	c	1/9 a is a * with same coverage
				*rbcL*	1051	1051	1	0	0.9983	*Alstonia scholaris*	c	
				*trnL*	1589	1589	1	0	0.9977	*Alstonia scholaris*	c	1/2 is a *
11	*Alyxia reinwardtii*	Blume	Apocyn.	ITS2	614	614	0.8	1.00E-171	0.9912	*Alyxia reinwardtii*	c	
				*matK*	1317	1317	0.95	0	0.9972	*Alyxia reinwardtii*	c	
				*rbcL*	1020	1020	0.96	0	1	*Alyxia reinwardtii*	c	1/2 is a * with higher coverage
				*trnL*	1524	1524	0.98	0	0.9929	*Alyxia grandis*	a *	
12	*Hoya diversifolia*	Blume	Apocyn.	ITS2	507	507	0.63	3.00E-139	1	*Hoya glabra*	a *	
				*matK*	1347	1347	1	0	1	*Hoya vitellinoides*	a *	
				*rbcL*	1051	1051	0.99	0	1	*Hoya pottsii*	a *	
				*trnL*	1539	1539	0.98	0	0.9988	*Hoya* sp.	a *	
13	*Rauvolfia serpentina*	(L.) Benth. ex Kurz	Apocyn.	ITS2	617	617	0.73	1.00E-172	1	*Rauvolfia serpentina*	c	
				*matK*	1380	1380	0.99	0	1	*Rauvolfia serpentina*	c	
				*rbcL*	1057	1057	0.99	0	1	*Rauvolfia serpentina*	c	
				*trnL*	1395	1395	0.89	0	0.9873	*Rauvolfia serpentina*	c	
14	*Aglaonema commutatum*	Schott	Ar.	ITS2	501	805	0.59	2.00E-137	0.9964	*Thunbergia coccinea*	i	
				*matK*	1384	1384	1	0	0.9974	*Aglaonema crispum*	a *	
				*rbcL*	1022	1022	0.97	0	1	*Aglaonema commutatum*	c	
				*trnL*	1650	1650	1	0	0.9989	*Aglaonema crispum*	a *	
15	*Trevesia burckii*	R.Br.	Arali.	ITS2	745	745	0.95	0	0.988	*Trevesia palmata*	a *	
				*matK*	1393	1393	1	0	1	*Trevesia palmata*	a *	
				*rbcL*	1048	1048	0.98	0	0.9982	*Brassaiopsis gracilis*	a *	
				*trnL*	1668	1668	0.99	0	0.9989	*Brassaiopsis ciliata*	a *	
16	*Cibotium barometz*	(L.) J.Sm.	Ciboti.	ITS2	348	858	0.75	3.00E-91	0.9896	*Cucumis sativus*	i	
				*rbcL*	965	965	0.94	0	0.9872	*Cyathea chinensis*	a **	
17	*Decalobanthus mammosus*	(Lour.) A.R.Simoes & Staples	Convolvul.	*rbcL*	1031	1031	0.97	0	0.9982	*Merremia peltata*	a *	
18	*Erycibe malaccensis*	C.B.Clarke	Convolvul.	ITS2	466	466	0.95	5.00E-127	0.8631	*Erycibe obtusifolia*	a *	
				*matK*	1389	1389	1	0	1	*Erycibe cochinchinensis*	a *	
				*rbcL*	1033	1033	0.96	0	1	*Erycibe* sp.	a *	
				*trnL*	1347	1347	0.93	0	0.9881	*Erycibe coccinea*	a *	
19	*Rhododendron macgregoriae*	F.Muell.	Eric.	ITS2	723	723	1	0	0.9658	*Rhododendron groenlandicum*	a *	
				*matK*	1369	1369	1	0	0.9908	*Rhododendron javanicum*	a *	
				*rbcL*	1027	1027	0.98	0	0.9912	*Rhododendron simsii*	a *	
				*trnL*	1629	1629	0.96	0	0.9955	*Rhododendron javanicum*	a *	
20	*Acalypha grandis*	Benth.	Euphorbi.	ITS2	272	272	0.35	1.00E-68	0.9808	*Acer tataricum* subsp. *theiferum*	i	
				*rbcL*	1062	1062	0.99	0	1	*Acalypha grisebachiana*	a *	
				*trnL*	1729	1729	1	0	0.9886	*Acalypha hispida*	a *	
21	*Euphorbia tirucalli*	L.	Euphorbi.	ITS2	617	617	0.71	1.00E-172	1	*Euphorbia tirucalli*	c	1/12 I with higher coverage
				*rbcL*	1046	1046	0.98	0	1	*Euphorbia rauhii*	a *	
22	*Millettia sericea*	(Vent.) Benth.	Fab.	ITS2	712	712	0.94	0	0.9571	*Millettia pulchra*	a *	
				*matK*	1332	1332	0.97	0	0.988	*Millettia pulchra*	a *	
				*rbcL*	1042	1042	0.97	0	0.9982	*Dahlstedtia pinnata*	a *	
				*trnL*	1543	1543	1	0	0.9819	*Millettia pinnata*	a *	
23	*Parkia timoriana*	(DC.) Merr.	Fab.	ITS2	593	593	0.71	2.00E-165	0.9909	*Parkia timoriana*	c	
				*matK*	1376	1376	0.98	0	0.996	*Parkia biglandulosa*	a *	
				*rbcL*	1000	1000	0.95	0	0.9927	*Magnoliophyta* sp.	i	
				*trnL*	1814	1814	0.99	0	0.999	*Parkia biglandulosa*	a *	
24	*Phanera fulva*	(Korth.) Benth.	Fab.	ITS2	475	475	0.68	7.00E-130	0.9477	*Bauhinia* sp.	a *	
				*rbcL*	1016	1016	0.96	0	0.9982	*Embryophyte environmental*	i	
				*trnL*	1404	1404	0.78	0	0.9974	*Phanera vahlii*	a **	
25	*Orthosiphon aristatus*	(Blume) Miq.	Lami.	ITS2	562	562	0.69	5.00E-156	1	*Orthosiphon aristatus*	c	
				*rbcL*	1042	1042	0.98	0	1	*Clerodendranthus spicatus*	a **	
26	*Premna serratifolia*	L.	Lami.	ITS2	422	422	0.99	9.00E-114	0.8495	*Premna microphylla*	a *	
				*rbcL*	1040	1040	0.97	0	1	*Premna serratifolia*	c	2/3 is a * with higher and lower coverage
27	*Vitex glabrata*	Gaertn.	Lami.	ITS2	651	651	0.91	0	0.9558	*Vitex carvalhoi*	a *	
				*matK*	1587	1587	1	0	0.9988	*Vitex glabrata*	c	
				*rbcL*	1050	1050	1	0	0.9982	*Vitex doniana*	a *	
				*trnL*	1411	1411	0.94	0	0.9923	*Vitex triflora*	a *	
28	*Cinnamomum rhynchophyllum*	Miq.	Laur.	*matK*	1375	1375	0.99	0	0.9987	*Cinnamomum camphora*	a *	
				*rbcL*	1055	1055	1	0	1	Cinnamomum dubium	a *	
				*trnL*	1587	1587	1	0	1	Cinnamomum pittosporoides	a *	
29	*Ficus deltoidea*	Jack	Mor.	ITS2	616	616	0.78	4.00E-172	1	*Ficus deltoidea*	c	
				*matK*	1380	1380	1	0	0.996	*Ficus* cf.	a *	
				*rbcL*	1051	1051	0.98	0	0.9983	*Ficus benjamina*	a *	
				*trnL*	1664	1664	0.99	0	0.9967	*Ficus carica*	a *	
30	*Myristica succedanea*	Blume	Myristic.	ITS2	185	185	0.17	2.00E-42	0.9231	*Rhodohypoxis milloides*	i	
				*matK*	1476	1476	0.92	0	0.9988	*Myristica fragrans*	a *	
				*rbcL*	1057	1057	1	0	1	*Horsfieldia amygdalina*	a *	4/11 is a **
				*trnL*	1371	1371	0.83	0	0.9987	*Myristica iners*	a *	
31	*Nepenthes ampullaria*	Jack	Nepenth.	*matK*	1375	1375	0.99	0	0.9973	*Nepenthes mapuluensis*	a *	
				*rbcL*	1042	1042	1	0	1	*Nepenthes mirabilis*	a *	
				*trnL*	1648	1648	1	0	0.9956	*Nepenthes mirabilis*	a *	
32	*Nepenthes gracilis*	Korth.	Nepenth.	*matK*	1371	1371	1	0	0.9973	*Nepenthes gracilis*	c	
				*rbcL*	1046	1046	1	0	1	*Nepenthes mirabilis*	a *	
				*trnL*	961	961	0.57	0	0.9962	*Nepenthes ampullaria*	a *	
33	*Nepenthes mirabilis*	(Lour.) Druce	Nepenth.	ITS2	857	857	1	0	0.9979	*Nepenthes reinwardtiana*	a *	
				*matK*	1371	1371	1	0	0.9973	*Nepenthes mapuluensis*	a *	
				*rbcL*	1038	1038	1	0	0.9965	*Nepenthes graciliflora*	a *	
				*trnL*	959	959	0.57	0	0.9943	*Nepenthes sanguinea*	a *	
34	*Nepenthes reinwardtiana*	Miq.	Nepenth.	ITS2	861	861	1	0	0.9979	*Nepenthes reinwardtiana*	c	
				*matK*	1376	1376	1	0	0.996	*Nepenthes reinwardtiana*	c	
				*rbcL*	1042	1042	0.98	0	0.9965	*Nepenthes mirabilis*	a *	
				*trnL*	948	948	0.57	0	0.9924	*Nepenthes alba*	a *	
35	*Acriopsis liliifolia* var. *liliifolia*	(J.Koenig) Ormerod	Orchid.	ITS2	394	394	0.94	2.00E-105	0.8428	*Cymbidium ensifolium*	a **	
				*matK*	1408	1408	1	0	0.9987	*Acriopsis* sp.	a *	
				*rbcL*	911	911	1	0	0.9824	*Acriopsis* sp.	a *	
				*trnL*	824	1591	0.91	0	0.9265	*Cymbidium erythraeum*	a **	
36	*Cymbidium aloifolium*	(L.) Sw.	Orchid.	ITS2	468	468	0.61	1.00E-127	0.9884	*Cymbidium aloifolium*	c	
				*matK*	1386	1386	1	0	0.9987	*Cymbidium aloifolium*	c	1/5 is a *
				*rbcL*	1048	1048	0.98	0	0.9982	*Cymbidium aloifolium*	c	1/4 is a *
				*trnL*	989	989	0.79	0	0.953	*Cymbidium wadae*	a *	
37	*Cymbidium ensifolium*	(L.) Sw.	Orchid.	ITS2	387	387	0.66	4.00E-103	0.9072	*Cymbidium goeringii*	a *	
				*matK*	1293	1293	0.99	0	0.9889	*Cymbidium longibracteatum*	a *	
38	*Dendrobium crumenatum*	Sw.	Orchid.	ITS2	577	577	0.7	2.00E-160	0.9968	*Dendrobium crumenatum*	c	
				*matK*	1400	1400	0.99	0	0.9961	*Dendrobium crumenatum*	c	
				*rbcL*	1038	1038	0.97	0	0.9982	*Dendrobium pseudotenellum*	a *	
39	*Dendrobium purpureum*	Roxb.	Orchid.	ITS2	481	537	0.86	2.00E-131	0.9005	*Dendrobium calcaratum*	a *	
				*matK*	1360	1360	1	0	0.9947	*Dendrobium faciferum*	a *	
				*rbcL*	1042	1042	0.98	0	0.9965	*Dendrobium aggregatum*	a *	
				*trnL*	562	998	0.98	8.00E-156	0.9814	*Dendrobium chrysanthum*	a *	
40	*Dendrobium salaccense*	(Blume) Lindl.	Orchid.	ITS2	627	627	0.79	2.00E-175	0.9914	Dendrobium haemoglossum	a *	
				*matK*	1382	1382	0.99	0	0.9987	*Dendrobium salaccense*	c	
				*rbcL*	1031	1031	1	0	1	*Dendrobium salaccense*	c	2/3 is a *
				*trnL*	1328	1328	0.81	0	0.9959	*Dendrobium salaccense*	c	
41	*Grammatophyllum speciosum*	Blume	Orchid.	ITS2	809	38152	1	0	1	*Raphanus raphanistrum* subsp. *landra*	i	
				*matK*	1378	1378	0.99	0	0.996	*Grammatophyllum papuanum*	a *	
				*rbcL*	1037	1037	0.97	0	0.9947	*Cymbidium faberi*	a **	
				*trnL*	568	1103	0.93	2.00E-157	0.9905	*Cymbidium serratum*	a **	
42	*Nervilia concolor*	(Blume) Schltr.	Orchid.	ITS2	828	828	1	0	1	*Cucumis sativus*	i	
				*rbcL*	1062	1062	0.99	0	1	*Nepenthes mirabilis*	i	
				*trnL*	1585	1585	1	0	0.9834	*Nervilia mekongensis*	a *	
43	*Nervilia plicata*	(Andrews) Schltr.	Orchid.	ITS2	721	721	0.88	0	0.9741	*Syzygium megacarpum*	i	
				*matK*	1413	1413	0.97	0	0.9987	*Nervilia plicata*	c	
				*rbcL*	1005	1005	0.94	0	1	*Nervilia plicata*	c	1/4 is a * with higher coverage
				*trnL*	1663	1663	0.99	0	0.9967	*Nervilia plicata*	c	
44	*Oberonia lycopodioides*	(J.Koenig) Ormerod	Orchid.	ITS2	398	398	0.88	1.00E-106	0.8765	*Oberonia caulescens*	a *	
				*matK*	1205	1205	0.93	0	0.9732	*Oberonia mucronata*	a *	
				*rbcL*	922	922	1	0	0.9921	*Ancistrochilus* sp.	a **	
				*trnL*	592	1078	0.91	2.00E-164	0.8734	*Liparis loeselii*	a **	
45	*Strongyleria pannea*	(Lindl.) Schuit., Y.P.Ng & H.A.Pedersen	Orchid.	ITS2	431	431	0.59	2.00E-116	0.959	*Mycaranthes pannea*	c	
				*matK*	1375	1375	1	0	0.996	*Mycaranthes pannea*	c	
				*rbcL*	1055	1055	1	0	0.9965	*Mycaranthes pannea*	c	
46	*Galearia filiformis*	(Blume) Boerl.	Pand.	ITS2	433	433	0.99	4.00E-117	0.8552	*Populus nigra*	i	
				*matK*	1393	1393	1	0	1	*Galearia filiformis*	c	
				*rbcL*	1042	1042	0.98	0	1	*Galearia filiformis*	c	
				*trnL*	1744	1744	1	0	0.9969	*Galearia filiformis*	c	
47	*Benstonea affinis*	(Kurz) Callm. & Buerki	Pandan.	ITS2	124	124	0.24	6.00E-24	0.8611	*Magnolia henryi*	i	
				*matK*	1397	1397	0.91	0	0.9935	*Pandanus oblatus*	a *	
				*rbcL*	1057	1057	1	0	1	*Pandanus adinobotrys*	a *	
				*trnL*	1705	1705	1	0	0.9989	*Pandanus baptistii*	a *	
48	*Phyllanthus oxyphyllus*	Miq.	Phyllanth.	ITS2	621	621	0.74	9.00E-174	0.9971	*Phyllanthus oxyphyllus*	c	1/2 is a * with higher coverage
				*matK*	1375	1375	1	0	0.9973	*Phyllanthus oxyphyllus*	c	
				*rbcL*	1059	1059	1	0	1	*Phyllanthus emblica*	a *	
				*trnL*	989	989	0.58	0	0.9945	*Phyllanthus emblica*	a *	
49	*Ardisia complanata*	Wall.	Primul.	ITS2	667	667	0.78	0	0.9973	*Ardisia dasyrhizomatica*	a *	
				*matK*	1574	1574	1	0	0.9931	*Ardisia mamillata*	a *	
				*rbcL*	1031	1031	0.99	0	0.9965	*Ardisia crenata*	a *	
				*trnL*	1483	1483	1	0	0.9951	*Ardisia dasyrhizomatica*	a *	
50	*Ardisia crenata*	Sims	Primul.	ITS2	617	617	0.74	1.00E-172	0.997	*Ardisia villosa*	a *	
				*matK*	1404	1404	0.88	0	0.9987	*Ardisia crenata*	c	
				*rbcL*	1048	1048	1	0	1	*Ardisia cornudentata* subsp. *morrisonensis*	c	1/2 is a *
				*trnL*	1476	1476	0.99	0	0.9988	*Ardisia affinis*	a *	
51	*Ventilago madraspatana*	Boerl.	Rhamn.	ITS2	206	316	0.45	1.00E-48	0.9444	*Hibiscus panduriformis*	i	
				*matK*	1347	1347	0.96	0	0.9973	*Ventilago leiocarpa*	a *	
				*rbcL*	1022	1022	0.96	0	0.9947	*Ventilago leiocarpa*	a *	
				*trnL*	1574	1574	1	0	0.9722	*Ventilago kurzii*	a *	
52	*Psychotria montana*	Blume	Rubi.	ITS2	398	398	1	8.00E-107	0.9744	*Psychotria camerunensis*	a *	
				*matK*	1376	1376	0.99	0	0.996	*Psychotria asiatica*	a *	
				*rbcL*	1029	1029	0.96	0	1	*Psychotria adenophylla*	a *	
				*trnL*	1504	1504	0.96	0	0.9826	*Psychotria asiatica*	a *	
53	*Lunasia amara*	Blanco	Rut.	ITS2	579	579	0.74	6.00E-161	0.9654	*Lunasia amara*	c	
				*matK*	1243	1243	0.88	0	0.9971	*Lunasia amara*	c	
				*rbcL*	1026	1026	0.97	0	0.9947	*Flindersia brayleyana*	a **	
				*trnL*	1668	1668	0.95	0	0.9946	*Lunasia amara*	c	
54	*Melicope lunu-ankenda*	(Gaertn.) T.G. Hartley	Rut.	ITS2	787	787	1	0	0.9823	*Melicope pteleifolia*	a *	
				*matK*	1408	1408	1	0	0.9987	*Melicope pteleifolia*	a *	
				*rbcL*	1031	1031	0.98	0	0.9965	*Melicope pteleifolia*	a *	
				*trnL*	1168	1168	1	0	0.9953	*Melicope grisea*	a *	
55	*Kadsura scandens*	(Blume) Blume	Schisandr.	ITS2	558	558	0.69	7.00E-155	0.9967	*Kadsura scandens*	c	
				*matK*	1376	1376	1	0	0.9947	*Kadsura philippinensis*	a *	
				*rbcL*	1050	1050	0.99	0	1	*Kadsura* cf.	a *	
				*trnL*	1635	1635	0.99	0	0.986	*Kadsura matsudae*	a *	
56	*Smilax calophylla*	Wall. ex A.DC.	Smilac.	ITS2	821	821	1	0	0.9933	*Phaseolus vulgaris*	I	
				*rbcL*	1048	1048	0.98	0	0.9982	*Smilax cocculoides*	a *	
57	*Smilax zeylanica*	L.	Smilac.	ITS2	274	274	0.35	3.00E-69	0.9809	*Acer tataricum* subsp. *theiferum*	i	
				*matK*	1371	1371	1	0	1	*Smilax ovalifolia*	a *	
				*rbcL*	1044	1044	0.98	0	1	*Smilax ocreata*	a *	
58	*Aquilaria hirta*	Ridl.	Thymelae.	ITS2	702	702	0.82	0	0.9948	*Aquilaria microcarpa*	a *	
				*matK*	1402	1402	1	0	0.9974	*Aquilaria malaccensis*	a *	
				*rbcL*	1057	1057	0.99	0	1	*Rauvolfia serpentina*	c	
				*trnL*	987	987	0.67	0	0.9945	*Aquilaria microcarpa*	a *	
59	*Amomum hochreutineri*	Valeton	Zingiber.	ITS2	616	616	0.79	4.00E-172	0.9884	*Sundamomum hastilabium*	a **	
				*rbcL*	1044	1044	0.98	0	1	*Amomum villosum* var. *xanthioides*	a *	
				*trnL*	1568	1568	0.98	0	0.9931	*Amomum fulviceps*	a *	
60	*Etlingera solaris*	(Blume) R.M.Sm.	Zingiber.	ITS2	656	656	0.89	0	0.9764	*Hornstedtia conica*	a **	
				*rbcL*	1053	1053	0.99	0	1	*Alpinia arundelliana*	a **	
				*trnL*	1622	1622	0.99	0	0.9955	*Etlingera yunnanensis*	a **	
61	*Meistera aculeata*	(Roxb.) Skornick. & M.F. Newman	Zingiber.	ITS2	592	592	0.72	7.00E-165	1	*Amomum aculeatum*	c	
				*rbcL*	1020	1020	0.96	0	1	*Amomum dallachyi*	a *	

Note: Result summary: c = correct, a *: ambiguous or correct in genus level, a **: ambiguous or correct in family level, i = incorrect.
